# COP9 signalosome is required for adipose tissue maintenance and metabolic health

**DOI:** 10.1016/j.jlr.2026.100977

**Published:** 2026-01-08

**Authors:** Hongyi Zhou, Shayantani Chakraborty, Xuelei Zhao, Neal L. Weintraub, Huabo Su, Weiqin Chen

**Affiliations:** 1 Department of Physiology, Medical College of Georgia at Augusta University, Augusta, GA, USA; 2 Vascular Biology Center at Medical College of Georgia at Augusta University, Augusta, GA, USA; 3 Department of Medicine, Medical College of Georgia, Augusta University, Augusta, GA, USA; 4 Department of Pharmacology and Toxicology, Medical College of Georgia, Augusta University, Augusta, GA, USA

**Keywords:** protein homeostasis, adipose tissue, energy homeostasis, obesity

## Abstract

Constitutive photomorphogenesis mutant 9 (COP9) signalosome (CSN) is composed of eight subunits (CSN1 through CSN8). It acts as an essential regulator of Cullin-RING-ubiquitin ligases (CRLs), which target critical cellular regulators for degradation via the ubiquitin (Ub) proteasome pathway. The role of CSN in adipose tissue development and function has not yet been studied. We sought to determine the role of CSN8, the smallest subunit of the CSN complex, in adipogenesis, adipose tissue maintenance, and metabolic balance. We first found that CSN8 level remained constant during adipogenesis and knocking down CSN8 by CRISPR/Cas9 did not impair adipocyte differentiation. Notably, mice with adipocyte-specific *Csn8* gene deletion (*Csn8*^*AKO*^) showed disrupted CSN holo-complex formation and Cullin deneddylation, leading to the loss of white and brown adipose tissue. *Csn8*^*AKO*^ mice displayed insulin resistance while maintaining glucose tolerance. They showed increased food intake and a trend toward higher energy expenditure but were cold-intolerant. Bulk RNA sequencing revealed that CSN deficiency caused significant remodeling of white and brown adipose tissues, characterized by adipocyte death and inflammation. Specifically, white and brown adipose tissues lacking CSN8 exhibited marked upregulation of apoptotic and pyroptotic cell death, which was associated with alterations in ubiquitination and proteasome activity. In addition, *Csn8*^*AKO*^ mice were protected from high-fat diet-induced adipose tissue expansion but developed notable hepatomegaly, steatosis, and insulin resistance. Taken together, our data highlights that CSN8/CSN is crucial for maintaining protein homeostasis in adipose tissue, promoting adipocyte survival, supporting adipose tissue maintenance, and overall metabolic health.

Adipose tissue is essential for energy metabolism and is the body's most adaptable tissue, constantly changing in size and number of adipocytes (1). White adipose tissue (WAT) serves as both an energy storage depot and a key endocrine organ that regulates overall energy balance. In contrast, brown adipose tissue (BAT) can convert chemical energy into heat, potentially offering a protective effect against obesity and its related metabolic diseases (2). Defects in adipocyte development or function can lead to metabolic conditions like insulin resistance and diabetes. A detailed understanding of the complex mechanisms of adipose tissue biology is crucial for treating diseases such as obesity and lipoatrophy.

The constitutive photomorphogenesis 9 (COP9) signalosome (CSN) is an evolutionarily conserved multiprotein complex essential for various biological responses, including the cell cycle, signal transduction, DNA damage repair, transcriptional regulation, cell survival, and differentiation (3, 4). Canonical CSN consists of eight subunits (CSN1 through CSN8), which are required for CSN holo-complex assembly (5). Most of the functions of CSN are purportedly involved in regulating the activity of Cullin-RING ligases (CRLs) and the subsequent ubiquitin-proteasome system (UPS)-mediated proteolysis of their targeted substrates (6). CSN forms complexes with and inactivates CRLs by removing a small ubiquitin-like modifier named neural precursor cell-expressed, developmentally downregulated 8 (NEDD8). Cullin neddylation is essential for the assembly and activation of the CRL, while Cullin deneddylation is crucial for disassembly and, thus, the dynamics of CRLs (3, 4). CSN5 contains isopeptidase activity, which removes NEDD8 from Cullins via deneddylation (7). Loss of one CSN subunit destabilizes other CSN subunits to varying degrees, resulting in reduced deneddylation activity and destabilization of substrate-recognizing adaptors in CRLs (8, 9). In addition, CSN recruits deubiquitinating enzymes, which remove ubiquitin from monoubiquitinated or polyubiquitinated substrates (3). CSN is also associated with different kinases, which phosphorylate the substrates of the UPS, thereby modulating protein stability (10). CSN and its variants CSN^CSN7A^ and CSN^CSN7B^ have been shown to influence adipogenesis (11, 12). During adipocyte differentiation, CSN subunits were reported to be regulated differently, with CSN6/8 levels decreasing and CSN7 levels increasing in mature adipocytes compared to preadipocytes (13). Knocking down CSN1 or CSN7A impaired adipogenesis (12, 14, 15), whereas overexpressing CSN7A accelerated it (16). However, the physiological role of CSN in the development and function of adipose tissue has never been investigated.

CSN8 is the smallest and the least conserved subunit of the CSN complex required for the structural integrity of CSN holo-complex and CUL deneddylation (17, 18). Free monomeric CSN8 is generally not detected under normal conditions; however, evidence suggests the existence of a low-abundance, small CSN8-containing complex separate from the main CSN complex (19). Conditional deletion of CSN8 during late T-cell development impairs the reactivation of quiescent T-cells in response to immune stimulation (20). Hepatic deficiency of CSN8 induces UPS impairment and Bim-mediated apoptosis of hepatocytes in murine liver (21, 22). Cardiac ablation of CSN8 impairs myocardial protein degradation by UPS and autophagic-lysosomal pathways, resulting in cardiac hypertrophy, heart failure, and premature death in mice (17, 23). However, the exact roles of CSN8 in adipose development and function remain to be defined.

In this study, we examined the role of CSN8/CSN in adipose tissue function by generating mice with CSN8 specifically deleted in adipocytes. Analyzing these mice under normal diet conditions and after they developed diet-induced obesity, along with in vitro experiments, showed that CSN is not necessary for adipogenesis but is vital for the survival of mature white and brown adipocytes at least in vivo. Our findings suggest that the COP9 signalosome plays a crucial role in regulating the mass of WAT and BAT, as well as overall energy balance, thereby enhancing our understanding of adipocyte biology and potentially opening new avenues for treating adipose-related disorders.

## Materials and methods

### Mice

*Csn8*^*f/f*^ mice (17) were bred with mice in which Cre expression is driven by the adiponectin promoter (JAX # 01083) to delete exons 4–6 of *Csn8* to generate adipocyte-specific *Csn8* knockout (*Csn8*^*AKO*^) mice. *Csn8*^*f/f*^ littermates served as wild-type controls (Ctrl). All mice, unless otherwise specified, were fed a chow diet (2018 Teklad Global 18% Protein Rodent Diet, Inotiv Inc.) and maintained under standard conditions with a controlled 12 h/12 h light/dark cycle at 21°C ambient temperature. For diet-induced obesity, 6-week-old mice were fed a high-fat diet (HFD, 60% fat by kcal; D12492, Research Diets Inc., New Brunswick, NJ). Body weight (BW) was measured weekly. Adipose tissues and liver were dissected and weighed and normalized to the BWs. Male mice were mainly used, with the main metabolic phenotypes investigated in female mice. Genotype-, age- and sex-matched mice were randomly assigned to treatment groups. The IACUC at Augusta University approved all animal experiments (IACUC File # 2012-0462).

### Cell culture, CRISPR/Cas9 gene targeting, lentivirus production and infection, adipocyte differentiation, triglyceride contents, and oil-red O staining

All cell culture reagents were obtained from Thermo Fisher Scientific Inc. (Pittsburgh, PA). Insulin, dexamethasone, isobutylmethylxanthine (IBMX), and puromycin were obtained from MilliporeSigma (St. Louis, MO). All cells were cultured at 37°C incubator with 5% CO2. Mouse embryonic fibroblasts were isolated from 12.5- to 14.5-day-old embryos from C57BL/6J mice and differentiated into adipocytes as described (24, 25). RAW264.7 (ATCC TIB-71) was cultured in Dulbecco’s modified Eagle’s medium (DMEM) containing 10% fetal bovine serum and 1% penicillin-streptomycin. The 3T3-L1 cells (ATCC CL-173) were maintained in DMEM containing 10% calf bovine serum (ATCC® 30–2030™) and 1% penicillin-streptomycin. Lentiviral particles expressing pLenti-CRISPR V2 empty vector or gRNA1 and gRNA2 against murine *Csn8* (GenScript, New Jersey, NJ) were produced in HEK293T cells as previously described (26). The two guide RNA (gRNA) sequences are: gRNA1, GAACCGAGCTCAAGCTGCCA; gRNA2, GCTAGAAGCTGACCGTACAC. Virus supernatants were combined with fresh DMEM complete medium at a 1:1 ratio to infect proliferating 3T3-L1 preadipocytes overnight in the presence of 8 μg/ml polybrene, respectively. Cells were then selected with 2 μg/ml puromycin for at least four days before being cultured to confluence for adipocyte differentiation following the standard hormone cocktail protocol as previously described (25). On the indicated days, cells were directly lysed in PBS containing 1% Triton X-100 to measure intracellular triglycerides (TGs) using an Infinity Triglyceride assay kit (Thermo Fisher Scientific). Data were normalized to the amount of cellular protein as determined using a Bradford protein assay (Bio-Rad Laboratories, Hercules, CA). Oil-red O staining was performed and photographed using a camera or a microscope, as described previously (25).

### Human/mouse adipose tissue fractionation and preparation of conditioned media from adipocytes

Deidentified human mediastinal adipose tissue was collected as surgical waste from patients undergoing cardiothoracic surgeries. All procedures involving human adipose tissue were approved by the Augusta University Biosafety Office (protocol # 1210). Human and mouse adipose tissues were digested with collagenase type IV at 37°C in a water bath, shaken at 100 rpm for 40–60 min. The digest was filtered through a 250-μm-pore-size mesh, followed by centrifugation at 100 g for 10 min to separate the stromal vascular fraction from the floating adipocytes. Mouse primary adipocytes were cultured in conditioned media (CM) (Phenol-red free DMEM with 2% FFA-free BSA, L-glutamine, 1% penicillin-streptomycin) at a 1:1 ratio (1 ml adipocytes/1 ml CM) for 18 h. CM were then collected as previously described (27) and used to culture RAW264.7 cells at a 1:1 dilution for 18 h.

### Histology, immunohistochemistry, and immunofluorescent staining

Tissue samples were fixed, processed, and stained with hematoxylin and eosin (H&E) for examination. For MAC2 immunohistochemistry, the sections were incubated overnight with anti-MAC2 (125401, BioLegend, RRID: AB_1134237), followed by detection using the ABC Vectastain Elite kit (Vector Laboratories) according to the manufacturer’s instructions. Immunofluorescent staining of PLIN1 was carried out in paraffin-embedded sections after deparaffinization, rehydration, and antigen retrieval. Sections were then incubated overnight with anti-PLIN1 (Cell Signaling Technology, Danvers, MA), followed by probing against F(ab')2-Goat anti-Rabbit IgG (H + L) Cross-Adsorbed Secondary Antibody, Alexa Fluor 594 (Thermo Fisher Scientific, Cat#A11072). Sections were then counterstained with 4′,6-Diamidino-2-phenylindole (DAPI, Sigma-Aldrich, D-9542), mounted. Fluorescent images were captured with a Fluorescence Microscope (BZ-X710, KEYENCE, Osaka, Japan).

### Measurement of blood parameters

Enzymatic assay kits were used to determine serum nonesterified fatty acids (NEFAs) (WAKO NEFA analysis kit (NEFA-HR(2); Wako Pure Chemical Industries), glycerol (free glycerol reagent, Cat#F6428, MilliporeSigma, Burlington, MA), triglycerides (Infinity Triglycerides Assay Kit, Cat#TR-22421, Thermo Fisher Scientific) and cholesterol (Infinity Cholesterol Assay Kit, Cat#TR-13421, Thermo Fisher Scientific). Plasma insulin (Millipore Sigma, Cat#EZRMI-13K) and leptin (Millipore Sigma, Cat#EZML-82K) levels were measured using enzyme-linked immunosorbent assay kits according to the manufacturer’s protocols.

### Tissue triglyceride contents, glucose, and insulin tolerance tests

Tissue TGs were extracted and dissolved in chloroform as previously described (28). A small aliquot (5–30 μl) was removed and dried. The TG concentration in this aliquot was determined using the Infinity Triglyceride Assay Kit and normalized to tissue weight. Glucose tolerance tests were performed in 6 h fasted chow diet-fed mice or 16 h fasted HFD-fed mice with intraperitoneally (*i*.*p*) injection of glucose at 1.5 g glucose/kg BW. Insulin tolerance tests were performed in 6-h fasted mice with an *i*.*p*. injection of human insulin (Novo Nordisk) at 0.75 U/kg for mice on a chow diet and 2.0 U/kg for mice on a HFD, respectively. Blood glucose levels were measured by a One-touch Ultra glucose meter before and at 15, 30, 60, and 120 min after glucose or insulin administration.

### Body composition, indirect calorimetry, and thermogenesis

Body composition, including fat and lean body masses, was assessed using nuclear magnetic resonance (NMR)-based Bruker Minispec mq10. Food intake, activity, oxygen consumption, and energy expenditure were assessed in a metabolic monitoring system (Comprehensive Lab Animal Monitoring System (CLAMS), Columbus Instruments, Columbus, OH) for 4 days (2 days of acclimation followed by 2 days of measurement) according to the manufacturer’s protocols. All measurements were expressed as normalized to BW. Locomotor activity was measured by counting the number of infrared beam breaks on the x- y- and z-axes during the measurement period. Experiments were conducted at ambient temperature (21˚C). The core (rectal) temperature of individually housed mice was measured using a thermocouple probe connected to a thermocouple thermometer (BAT-12, Physitemp, Clifton, NJ) at 22°C ambient and at 1, 2 and 3 h after being placed at 4°C in the absence of food.

### Measurement of proteasome activity and protein carbonylation

We used the Proteasome Activity Fluorometric Assay (Cat #J4110, UBPBio, Dallas, TX) according to the manufacturer’s instructions to assess chymotrypsin-, trypsin-, and caspase-like activity. In each assay, we utilized MG132 (MilliporeSigma) controls for each sample to determine the specific proteasome activity (Δslope) over time. Protein oxidation was assessed using the OxyBlot Protein Oxidation Detection Kit (S7150; MilliporeSigma, Billerica, MA) according to the manufacturer's instructions.

### RNA sequencing and analysis

Total RNA was extracted by TRIzol™ (Thermo Fisher Scientific) from subcutaneous adipose tissues of 3-month-old Ctrl and *Csn8*^*AKO*^ mice (male, 4 h fast, n = 4) and brown adipose tissues of 4-week-old Ctrl and *Csn8*^*AKO*^ mice (male, 4 h fast, n = 5). Differential gene expression analysis was performed using bulk RNA-seq at the Genome Technology Access Center at Washington University. The library was prepared using a RiboErase rRNA removal kit (Kapa Biosystems). Complementary DNA (cDNA) was generated using SuperScript III RT enzyme (Life Technologies). cDNA was blunt-ended, had an A base added to the 3′ ends, and then had Illumina sequencing adapters ligated to the ends. Ligated fragments were then amplified for 12–15 cycles using primers incorporating unique dual index tags. Fragments were sequenced on an Illumina NovaSeq X Plus using paired end reads extending 150 bases. Basecalls and demultiplexing were performed with Illumina’s bcl2fastq software with a maximum of one mismatch in the indexing read. RNA-seq reads were then aligned to the Ensembl release 101 primary assembly with STAR version 2.7.9a1. Gene counts were derived from the number of uniquely aligned unambiguous reads by Subread:featureCount version 2.0.32. Isoform expressions of known Ensembl transcripts were quantified with Salmon version 1.5.23. We performed differential gene expression analysis of count data using the R Package DESeq2 (1.36.0). We used a Benjamini–Hochberg false discovery rate-adjusted *P* value less than or equal to 0.05 to select differentially expressed transcripts. Metascape was used to further analyze and annotate Kyoto Encyclopedia of Genes and Genomes (KEGG) pathways. SRplot was used to draw an enrichment bubble plot.

### RNA isolation and real-time quantitative PCR

Total RNA was isolated using TRIzol reagent (Thermo Fisher Scientific, Cat#15-596-018) according to the manufacturer's guidelines. The quality and quantity of the extracted RNA were assessed using a BioTek Take 3 spectrophotometer. For reverse transcription, we used MLV-V reverse transcriptase coupled with random primers (Thermo Fisher Scientific). Real-time quantitative RT-PCR was performed using SYBR Green Dye on the AriaMX (Agilent, Santa Clara, CA). Gene expression levels were normalized to two housekeeping genes (*Rplp0* and/or *Ppia*) based on the Genorm Algorithm (RRID:SCR_006763). Data were expressed as fold changes relative to control tissue or cells. All tissue gene expression studies were performed in mice after 4 h fast. Supplemental Table S1 lists the RT-PCR primer sequences for genes that were analyzed.

### Western blots

Total proteins were extracted by homogenizing tissues or cells in a lysis buffer containing 25 mM Tris–HCl (pH 7.4), 150 mM NaCl, 2 mM EDTA, 1% Triton X-100%, and 10% glycerol with freshly added protease inhibitor cocktail (MilliporeSigma) and phosphatase inhibitor (Thermo Fisher Scientific). The protein concentrations were determined using the Bradford protein assay (Bio-Rad). Standard Western immunoblotting procedures were carried out to analyze the proteins. Chemiluminescent substrate ECL (MilliporeSigma) was used for blot development with an A600 Imager (Cytiva, Marlborough, MA). iQTL software (Cytiva, Marlborough, MA) and ImageJ were utilized for the quantification of the results. The antibodies used are listed in Supplemental Table S2.

### Quantification and statistical analysis

Quantitative data were presented as means ± standard error of the mean (SEM). All animal experiments were carried out in at least two independent cohorts, except for the RNA-Seq and HFD-induced obesity experiments. Sample sizes were based on retrospective data and an R2 value of 0.8 for the functional assays corresponding with a value of n = 8, a = 0.05 and 80% power, assuming a linear regression model with two prior covariates. Statistical significance was assessed with GraphPad Prism 10.1.3 using either an unpaired *t* test or multiple unpaired t-tests with the Holm–Sidak method or two-way analysis of variance (ANOVA) with Tukey’s multiple comparison test, as dictated by the experiments. Shapiro–Wilk or Kolmogorov–Smirnov tests were performed to assess normality before statistical analysis. Detailed statistical tests and corresponding sample sizes (n) are provided in the respective figure legends. A *P* value of less than 0.05 was considered statistically significant.

## Results

### CSN8 is dispensable for white adipocyte differentiation

**Fig. 1:**
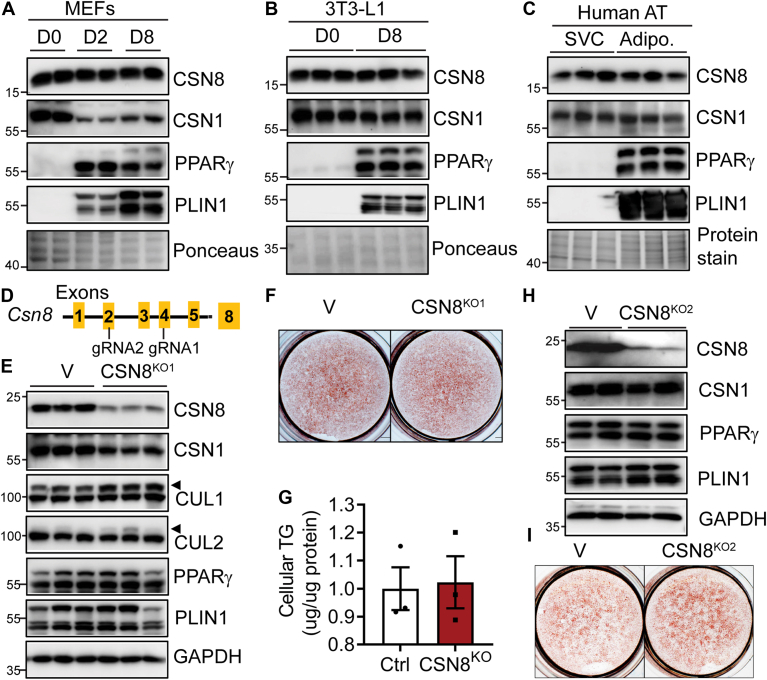
CSN8 is dispensable for adipocyte differentiation. A–B: Protein expression during the adipocyte differentiation of MEFs at days (D) 0, 2, and 8 after induction of adipocyte differentiation. B: Protein expression in D0 (before induction) and D8 mature 3T3-L1 adipocytes. C: CSN8 expression in stromal vascular cells (SVCs) and mature adipocytes fractionated from the human mediastinal adipose biopsy. D: CRISPR/Cas9 strategy indicating the targeted exons of gRNA1 and gRNA2 used to delete murine CSN8. E: 3T3-L1 cells were infected with lentiviruses expressing empty vector (V) or gRNA1 against the murine *Csn8* gene to knock down CSN8 (KO1). Cells were differentiated using a standard protocol, and protein expression was examined in D8 mature adipocytes. Arrowhead indicates neddylated CULs. F–G: Oil-red O staining and intracellular triglyceride content quantification in D8 adipocytes. H–I: Western blot and Oil-red O staining in D8 mature adipocytes differentiated from V and CSN8 KO2 cells generated from gRNA2 against the murine *Csn8*. Data are from three independent experiments. MEF, mouse embryonic fibroblast.

We first examined whether the expression of CSN8 is altered during adipocyte differentiation. CSN8 did not exhibit significant changes at the protein level during the differentiation of white adipocytes from mouse embryonic fibroblasts. However, a reduction in CSN1 was noted in differentiating (2 days after induction) and mature (8 days after induction) adipocytes (Fig. 1A). Expression of both CSN8 and CSN1 was similar in 3T3-L1 preadipocytes (day 0) versus differentiated (day 8) mature adipocytes (Fig. 1B), and in stromal vascular cells (SVCs) and mature adipocytes fractionated from human adipose tissue biopsy (Fig. 1C). Therefore, the expression of components of the CSN complex, particularly CSN8, was not perturbed during adipocyte differentiation.

To investigate whether CSN8 is involved in adipocyte differentiation, we applied the CRISPR/Cas9 strategy to delete CSN8 using two gRNAs in 3T3-L1 preadipocytes, respectively, and then subjected cells to hormone cocktail-induced adipogenesis (Fig. 1D). In mature adipocytes 10 days after differentiation, we found that CSN8 expression was markedly reduced by gRNA1 compared to vector-infected control cells (Fig. 1E). Knockdown of CSN8 led to a reduction in the expression of other CSN subunits, such as CSN1, suggesting impaired CSN complex formation in CSN8^KO1^ adipocytes (Fig. 1E). Subsequently, there was increased neddylation of Cullin 1 (CUL1) and CUL2, which exhibited a slower migration rate than the corresponding native forms, indicating that the Cullin deneddylation activity was compromised in CSN8^KO1^ adipocytes (Fig. 1E). However, knockdown of CSN8 did not impact adipocyte differentiation, as indicated by similar expression of adipocyte marker proteins (PPARγ and PLIN1) (Fig. 1E) and TG accumulation assessed through oil-red O staining and cellular TG content (Fig. 1F–G) between Control and CSN8^KO1^ cells. Similar results were also observed in gRNA2-generated CSN8^KO2^ 3T3-L1 cells (Fig. 1H–I). Therefore, CSN8 and its associated COP9 signalosome activity are dispensable for adipocyte differentiation.

### Adipose-specific deletion of CSN8 reduces adiposity in mice

**Fig. 2:**
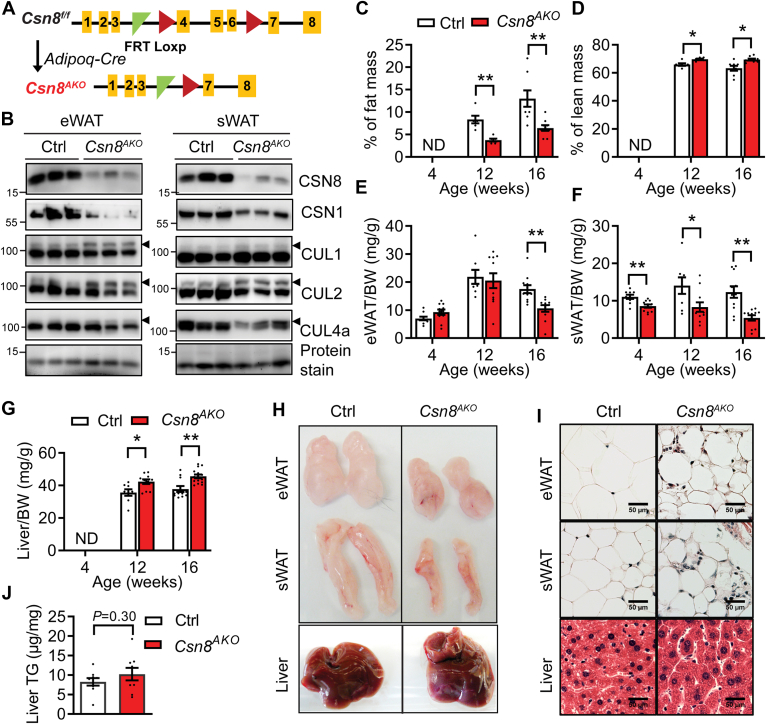
Adipose-specific loss of CSN8 reduces adiposity in male mice. A: Strategy depicting the generation of *Csn8*^*f/f*^; *AdipoqCre*^-^ (Ctrl) and *Csn8*^*f/f*^; *AdipoqCre*^*+*^ (*Csn8*^*AKO*^) mice. B: Western blot confirming the depletion of CSN8, the expression of CSN1 and neddylation of CULs in eWAT and sWAT, respectively. Arrowhead indicates neddylated CULs. (C) % of fat mass, (D) % of lean mass, (E) eWAT, (F) sWAT, and (G) liver weights as normalized to body weights (BW) in 4-, 12-, and 16-week-old male Ctrl and *Csn8*^*AKO*^ mice. ND: not determined. n = 6–13/group. Multiple unpaired *t* test with the Holm–Sidak method. H: Representative tissue morphology after dissection and (I) representative hematoxylin & eosin staining of eWAT, sWAT, and liver from 16-week-old male Ctrl and *Csn8*^*AKO*^ mice. Scale bar = 50 μm. J: Liver triglyceride contents in 6-month-old male Ctrl and *Csn8*^*AKO*^ mice. n = 9/group. Unpaired *t* test. WAT, white adipose tissue; eWAT, epididymal WAT; sWAT, subcutaneous WAT. ∗*P* < 0.05; ∗∗*P* < 0.005 versus Ctrl.

Since CSN8 is dispensable for adipocyte differentiation, we next generated adipocyte-specific *Csn8* knockout mice using the Adiponectin-Cre driver strain (*Csn8*^*AKO*^) to evaluate the role of CSN8 in regulating mature adipose function (Fig. 2A). CSN8 was significantly deleted in epididymal white adipose tissue (eWAT) and subcutaneous WAT (sWAT) of *Csn8*^*AKO*^ mice, respectively (Fig. 2B). We also observed reduced expression of other CSN subunits, such as CSN1 (Fig. 2B). Furthermore, eWAT and sWAT in *Csn8*^*AKO*^ mice displayed an increase of neddylated CUL1, CUL2, and CUL4A (Fig. 2B), indicating that the Cullin deneddylation activity was compromised in *Csn8*-deficient adipose tissue. 4 to 16-week-old male *Csn8*^*AKO*^ mice maintained similar BWs compared to *Csn8*^*f/f*^ Control (Ctrl) mice (Supplemental Fig. S1A). However, by 12 weeks of age, there was about a 51% reduction of total body fat mass and a 9.8% increase in lean mass in male *Csn8*^*AKO*^ mice, respectively, compared with their wildtype counterparts (Fig. 2C–D). Such changes were further exacerbated in 16-week-old male *Csn8*^*AKO*^ mice (Fig. 2C–D). Interestingly, loss of eWAT was only evident starting at 16 weeks of age in male *Csn8*^*AKO*^ mice based on either the absolute mass or after normalization to BW (Supplemental Fig. S1B and Fig. 2E, respectively). By contrast, *Csn8*^*AKO*^ sWAT exhibited a more obvious reduction starting from as early as 4 weeks old, with an almost 50% reduction in sWAT mass by 16 weeks old (Supplemental Fig. S1C and Fig. 2F). Although the absolute liver masses only trended higher (Supplemental Fig. S1D), liver weights normalized to BWs were significantly increased in 12 and 16-week-old male *Csn8*^*AKO*^ mice (Fig. 2G). Morphological analyses confirmed reduced eWAT and sWAT as well as increased liver sizes in 16-week-old male *Csn8*^*AKO*^ mice (Fig. 2H). Histological analysis revealed changes in adipocyte size and an increase in stromovascular cells in the eWAT and sWAT of 16-week-old male *Csn8*^*AKO*^ mice (Fig. 2I). Despite an increase in their liver sizes, there were no apparent differences in liver histology in *Csn8*^*AKO*^ mice (Fig. 2I). Further analysis identified no differences in liver TG contents even between 6-month-old male Ctrl and *Csn8*^*AKO*^ mice (Fig. 2J). In addition, even though there were no significant differences in BWs (Supplemental Fig. S2A), 16-week-old female *Csn8*^*AKO*^ mice demonstrated about 25% loss of fat mass with maintained lean mass (Supplemental Fig. S2B and C, respectively). Upon dissection, 16-week-old female mice exhibited a 55% reduction in gonadal WAT (gWAT) and a 67% loss in sWAT, together with a 24% increase in liver masses (Supplementary Fig. S2D–E). These data suggest that *Csn8*^*AKO*^ mice exhibit a lean phenotype in both male and female mice, indicating an essential role of CSN8 in maintaining adipose tissue mass.

### Mice with adipose-specific deletion of CSN8 exhibit insulin resistance and altered energy homeostasis

**Fig. 3:**
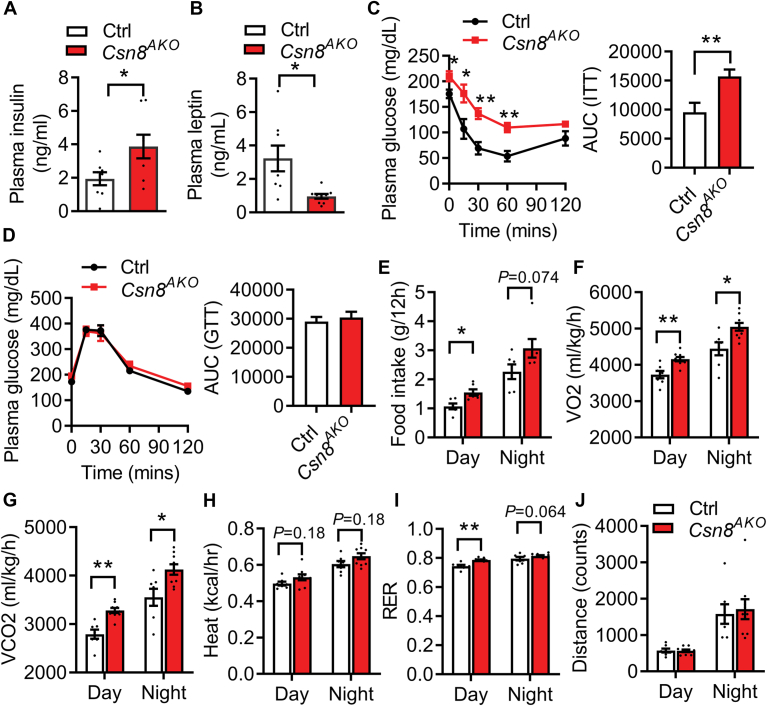
Mice with adipose-specific deletion of CSN8 display insulin resistance and altered energy homeostasis. (A) Plasma insulin and (B) leptin levels in 4 h fasted 16-week-old male Ctrl and *Csn8*^*AKO*^ mice (n = 8–9/group). Unpaired *t* test. (C) Insulin tolerance tests and area under the curve (AUC), (D) glucose tolerance tests and AUC. Multiple unpaired *t* test with the Holm-Sidak method. (E) Food intake, (F) O2 consumption (VO2), (G) CO2 production (VCO2), (H) heat production, (I) respiratory exchange ratio (RER), and (J) distance as measured by counts of beam breaks during day and night periods. 16-week-old male Ctrl and *Csn8*^*AKO*^ mice were used. n = 7–9/group. Two-way ANOVA with Tukey’s multiple comparison test. ∗*P* < 0.05; ∗∗*P* < 0.005.

Reduced adipose mass is linked to altered nutritional and energy homeostasis. We first found no significant differences in the fasting plasma levels of TG, cholesterol, and NEFA, except for slightly reduced plasma glycerol levels in *Csn8*^*AKO*^ mice fed a normal chow diet (Table 1). However, male *Csn8*^*AKO*^ mice manifested higher plasma insulin levels (Fig. 3A). Their leptin levels were also reduced compared to Ctrl mice, in line with their reduced body fat mass (Fig. 3B). Not surprisingly, male *Csn8*^*AKO*^ mice displayed insulin resistance, but they were still able to maintain similar glucose tolerance as compared to Ctrl mice (Fig. 3C-D, respectively). Female *Csn8*^*AKO*^ mice also displayed significant insulin resistance, accompanied by maintained glucose tolerance, confirming the development of a prediabetic phenotype only (Supplementary Fig. S2F–G, respectively). Male *Csn8*^*AKO*^ mice were hyperphagic, especially during the daytime (Fig. 3E). These mice also exhibited higher oxygen consumption and CO2 production during both day and night (Fig. 3F–G). There was a tendency for higher heat production in *Csn8*^*AKO*^ mice (Fig. 3H). In line with higher food intake, male *Csn8*^*AKO*^ mice exhibited a higher respiratory exchange ratio, particularly during the daytime (Fig. 3I). There was no difference in activity levels between the two genotypes (Fig. 3J). These data suggest that deletion of CSN8/CSN in adipocytes disrupts whole-body insulin sensitivity and energy balance.

**Table 1:** Plasma parameters in male Ctrl and *Csn8*^*AKO*^ mice fed with a normal chow diet or a high-fat diet

Diet	CD		HFD	
Genotype	Ctrl (n = 8)	*Csn8*^*AKO*^ (n = 9)	Ctrl (n = 5)	*Csn8*^*AKO*^ (n = 9)
TG (mg/dl)	43.4 ± 2.7	44.2 ± 3.2	36.1 ± 3.2	37.5 ± 2.7
TC (mg/dl)	81.7 ± 3.3	85.3 ± 2.4	101.1 ± 8.4	99.3 ± 4.6
NEFA (mM)	1.43 ± 0.12	1.77 ± 0.15	0.95 ± 0.14	1.01 ± 0.1
Glycerol (mg/dl)	35.5 ± 2.0	28.1 ± 1.8^a^	38.9 ± 3.6	25.0 ± 1.4^b^

NEFA, nonesterified fatty acid; TC, total cholesterol; TG, triglyceride.

Plasma biochemistry was analyzed in 4-h fasted normal chow diet (CD)-fed 16-week-old male Ctrl and *Csn8*^*AKO*^ mice, and Ctrl and *Csn8*^*AKO*^ mice fed with high-fat diet (HFD) for 10 weeks starting from 6 weeks of age. Data were presented as means ± SEM.

a *P* < 0.05.

b *P* < 0.005 versus Ctrl mice under the same diet.

### CSN8-deleted WATs show reduced metabolic signaling and increased inflammation

**Fig. 4:**
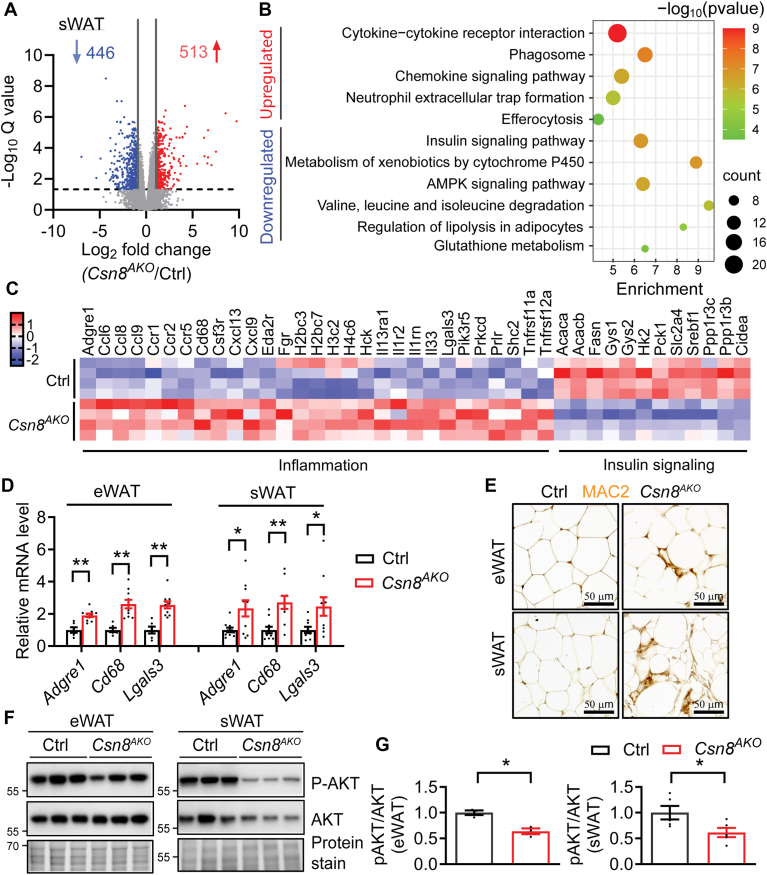
CSN8 deletion induces adipose tissue inflammation and insulin resistance. A: Volcano plot for differentially expressed genes (DEGs) in the sWAT of 12-week-old male Ctrl and *Csn8*^*AKO*^ mice (n = 4). The *y*-axis denotes −log_10_*P*_*adj*._ (Q) values, while the *x*-axis shows log_2_ fold change values. DEGs; 1≤Log_2_FC ≤ −1. *P*_*adj*._<0.05. B: Bubble graph illustrates the upregulated and downregulated KEGG pathways filtered by DEGs in *Csn8*^*AKO*^ sWAT. The *x*-axis represents enrichment, while the color of the circle corresponds to the -log_10_*P* value, as indicated by the color bar. The size of the bubble represents the number of genes enriched in each pathway. Selective pathways indicated. C: Heatmaps showing the differential expression of inflammatory genes involved in cytokine-cytokine receptor interactions, chemokine signaling pathway, neutrophil extracellular trap pathway, and insulin signaling genes. D: RT-PCR of selective inflammatory genes in eWAT and sWAT of 16-week-old Ctrl and *Csn8*^*AKO*^ male mice. n = 6–8/group. Multiple unpaired *t* test with the Holm–Sidak method. E: Representative images from immunohistochemistry of MAC2 (gene name *Lgals3*) in eWAT and sWAT of a total of three 16-week-old male Ctrl and *Csn8*^*AKO*^ male mice. F–G: Western blot and quantifications of basal insulin-mediated AKT phosphorylation in the eWAT and sWAT of 4 h fasted 16-week-old male Ctrl and *Csn8*^*AKO*^ male mice. n = 3–5/group. Unpaired *t* test. ∗*P* < 0.05; ∗∗*P* < 0.005. KEGG, Kyoto Encyclopedia of Genes and Genomes; eWAT, epididymal white adipose tissue; sWAT, subcutaneous WAT.

To explore the specific mechanisms by which CSN8 affects WAT remodeling, we analyzed differential gene expression using bulk RNA-seq in sWAT from male Ctrl and *Csn8*^*AKO*^ mice at 12 weeks of age, a relatively young age at which *Csn8*^*AKO*^ mice demonstrated significant sWAT loss. We identified 446 genes that were markedly downregulated and 513 genes that were significantly upregulated (Fig. 4A). A KEGG analysis revealed enrichment for expression of genes involved in cytokine-cytokine receptor interaction, chemokine signaling pathway, neutrophil extracellular trap formation, as well as phagosome and efferocytosis pathways. In contrast, genes involved in insulin signaling pathway, cytochrome P450 pathway, AMPK signaling pathway, amino acid metabolism, regulation of lipolysis, and glutathione metabolism were significantly downregulated in the sWAT of *Csn8*^*AKO*^ mice (Fig. 4B). Heatmaps showed an increase in inflammatory genes (Fig. 4C). Upregulation of macrophage marker genes, including *Adgre1*, *Cd68*, and *Lgals3*, was confirmed by RT-PCR in both eWAT and sWAT of 16-week-old *Csn8*^*AKO*^ mice (Fig. 4D). Using immunohistochemistry to detect MAC2 (encoded by *Lgals3*), we detected enhanced staining in both eWAT and sWAT of *Csn8*^*AKO*^ mice, indicating increased presence of infiltrating macrophages (Fig. 4E). In line with reduced insulin signaling in *Csn8*^*AKO*^ eWAT, insulin resistance was observed at baseline, as shown by decreased pAKT signaling with preserved AKT expression (Fig. 4F, G). In *Csn8*-deficient sWAT, basal AKT expression was already reduced, and the p-AKT/AKT ratio was lower, pointing to insulin resistance (Fig. 4F, G). Overall, these findings suggest that deleting CSN8 in adipose tissue results in inflammation associated with insulin resistance.

### Upregulation of apoptosis and pyroptosis pathways in *Csn8*-deleted WATs

**Fig. 5:**
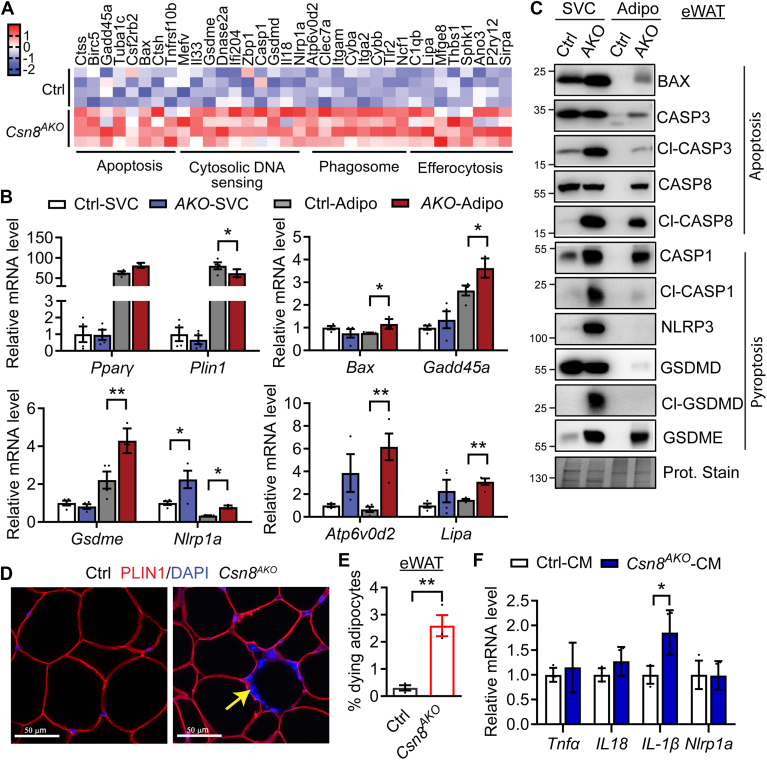
CSN8 deficiency causes adipose cell apoptosis and pyroptosis. A: Heatmap displaying the differential expression of genes involved in apoptosis, cytosolic DNA sensing, phagosome, and efferocytosis in the sWAT of 12-week-old Ctrl and *Csn8*^*AKO*^ male mice (n = 4) (DEGs cutoff: 0.75≤Log_2_FC ≤ −0.75, *P*_*adj*._<0.05). B: eWAT from 16-week-old control (Ctrl) and *Csn8*^*AKO*^ (AKO) male mice was fractionated into stromal vascular cells (SVCs) and adipocytes (Adipo) fractions. The expression of adipocyte marker genes (*Pparγ*, *Plin1*) and selective genes involved in apoptosis (*Bax*, *Gadd45a*), cytosolic DNA sensing (*Gsdme*, *Nlrp1a*), phagosome (*Atp6v0d2*), and efferocytosis (*Lipa*) was confirmed by RT-PCR. n = 4/group. Each group represents data combined from three mice. Gene expression in Ctrl-SVC was normalized to 1. Two-way ANOVA with Tukey’s multiple comparison test. C: Western blot in SVCs and Adipo fractionated from eWAT of 16-week-old male Ctrl and *Csn8*^*AKO*^ mice. Each blot includes samples combined from three animals. Representatives of three independent experiments are shown. D: Representative images of immunofluorescent staining of PLIN1 counter-stained with DAPI in eWAT of 16-week-old male Ctrl and *Csn8*^*AKO*^ mice. E: Quantitative analysis to determine the % of dead adipocytes calculated as (the number of PLIN1-negative adipocytes surrounded by DAPI-stained nuclei divided by the number of total adipocytes) x 100. Ten random images were quantified per animal and n = 3 per genotype. Unpaired *t* test. F: Inflammatory gene expression in RAW264.7 macrophages after 18 h treatment with conditioned media (CM) collected from cultured primary adipocytes isolated from eWAT of 16-week-old male Ctrl and *Csn8*^*AKO*^ mice. n = 4 per genotype. Two independent experiments. Multiple unpaired *t* test with the Holm–Sidak method. ∗*P* < 0.05; ∗∗*P* < 0.005. eWAT, epididymal white adipose tissue; sWAT, subcutaneous WAT; DAPI, 4’,6-Diamidino-2-phenylindole.

Since adipose inflammation is associated with adipocyte cell death, we next conducted detailed KEGG pathway analyses based on differentially expressed genes (0.75 ≤ Log2FC ≤ −0.75, *P*_adj_. < 0.05) identified from our bulk-RNA-Seq of sWAT. We found that pathways involved in apoptosis and cytosolic DNA sensing were upregulated in male *Csn8*^*AKO*^ sWAT (Fig. 5A). This is aligned with the increased expression of phagosome and efferocytosis pathway genes involved in the clearance of dying cells (Fig. 5A). We then investigated whether the decreased adiposity in *Csn8*^*AKO*^ mice could be due to enhanced adipocyte death. Given that adipose tissue comprises various cell types, we fractionated eWAT into SVCs and adipocyte-enriched fractions to specifically assess changes in cell death pathways. The expression of adipocyte marker genes (such as *Pparγ* and *Plin1*) was highly enriched in the adipocyte fractions isolated from eWAT from both Ctrl and *Csn8*^*AKO*^ mice. We observed no differences in the mRNA expression of *Pparγ* but a slight reduction of *Plin1* in *Csn8*^*AKO*^ adipocytes compared with Ctrl adipocytes (Fig. 5B). Consistent with increased apoptosis, there was a significant rise in the mRNA levels of proapoptotic genes, including *Bax* (∼1.5-fold) and *Gadd45a* (∼1.4-fold), specifically in *Csn8*^*AKO*^ adipocytes compared to Ctrl adipocyte fractions (Fig. 5B). Cytosolic DNA sensing triggers a cascade of events, including inflammasome activation and GSDMD/GSDME-mediated pore formation, ultimately leading to pyroptosis and a robust inflammatory response (29). The mRNA expression of the pyroptotic gene *Gsdme* was increased in *Csn8*^*AKO*^ adipocytes. In contrast, the expression of *Nlrpl1a* was increased in both SVCs and adipocyte fractions of *Csn8*^*AKO*^ mice (Fig. 5B). Consequently, the expressions of the selective phagocytic gene *Atp6v0d2* and efferocytosis-related gene *Lipa* trended higher in *Csn8*^*AKO*^ SVCs and were significantly elevated in *Csn8*^*AKO*^ adipocytes (Fig. 5B).

We further examined the presence of apoptosis and pyroptosis in the fractionated SVCs and adipocytes at the protein level. Notably, BAX levels were elevated in SVCs and adipocytes from eWAT of *Csn8*^*AKO*^ mice (Fig. 5C). Both CASP-3/8 and their cleaved/activated forms were significantly increased in the adipocyte-enriched fractions from *Csn8*^*AKO*^ eWAT (Fig. 5C), indicating adipocyte apoptosis in the absence of CSN8. Interestingly, the upregulation of cleaved CASP3 and cleaved CASP8 was more prominent in the SVCs from *Csn8*^*AKO*^ eWAT, suggesting their active roles in detecting and removing dead adipocytes (Fig. 5C). In addition to apoptosis, protein levels of CASP1, NLRP3, and GSDME were markedly higher in the SVCs from *Csn8*^*AKO*^ eWAT. Importantly, increased cleavage of CASP1 and cleaved-GSDMD—hallmarks of pyroptosis—were observed in *Csn8*^*AKO*^ SVCs, indicating inflammatory cell death (Fig. 5C). We also stained for the adipocyte-specific lipid droplet binding protein PLIN1 to detect PLIN1-negative dying adipocytes (30). Indeed, there was a significant increase in PLIN1-negative dead adipocytes in the eWAT of *Csn8*^*AKO*^ mice compared to Ctrl mice (Fig. 5D-E). Similarly, reduced *Plin1* expression along with increased expression of genes related to pyroptosis, phagocytosis, and efferocytosis (*Gsdme*, *Nlrp1a*, *Atp6v0d2*) were observed in adipocytes isolated from sWAT of *Csn8*^*AKO*^ mice (Supplemental Fig. S3A–B). In addition, levels of apoptotic and pyroptotic marker proteins were also elevated in the *Csn8*^*AKO*^ adipocytes from sWAT (Supplemental Fig. S3C). However, in vitro differentiated CSN8^KO1^ adipocytes did not undergo apoptosis or pyroptosis (Supplemental Fig. S4). Nevertheless, when macrophage cells were treated with conditioned media from isolated primary *Csn8*^*AKO*^ adipocytes, they showed increased interleukin 1β (IL1β) expression and a trend toward higher IL18 levels, indicating a possible role of *Csn8*^*AKO*^ adipocytes in promoting adipose inflammation thus inflammatory cell death in vivo (Fig. 5F). Collectively, our data suggest that CSN8 deletion in adipocytes induces adipocyte apoptosis and inflammatory pyroptosis associated with the activation of phagosome and efferocytosis to remove dead cells in both eWAT and sWAT.

### CSN8 deletion in adipocytes increases oxidative stress, disrupts protein homeostasis by perturbing protein ubiquitination and the activity of the proteasome

**Fig. 6:**
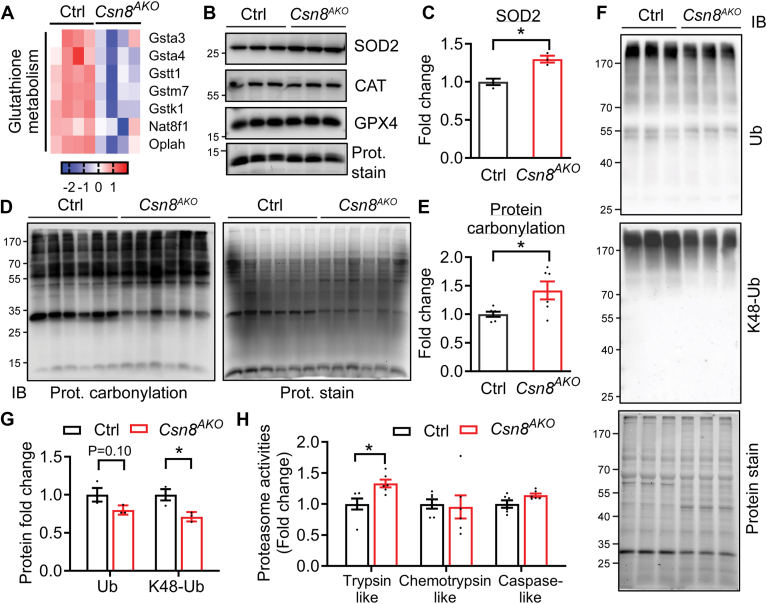
CSN8 deletion induces oxidative stress, reduces protein ubiquitination associated with increased proteasome function. A: Heatmap displaying the differential expression of genes involved in glutathione metabolism in the sWAT of 12-week-old Control (Ctrl) and *Csn8*^*AKO*^ male mice (n = 4) (DEGs cutoff: 1.0≤Log_2_FC ≤ −1.0, *P*_*adj*._<0.05). B-C: Western blot and quantitative analyses of antioxidant proteins (n = 3). Unpaired *t* test. D-E: Measurements of dinitrophenylhydrazine (DNP) derivatives indicate the overall carbonyl groups introduced into the protein side chains by oxidative modification in the eWAT of 16-week-old male Ctrl and *Csn8*^*AKO*^ mice. Data were quantified and normalized to the protein signals in the stain-free gel with Ctrl eWAT normalized to 1. n = 6/group. Unpaired *t* test. F–G: Western blot and quantitative analyses of ubiquitinated and K48-ubiquitinated proteins after normalization to the protein-stained signals in the isolated adipocytes from eWAT of 16-week-old male Ctrl and *Csn8*^*AKO*^ mice (n = 3). Unpaired *t* test. H: Proteasome activity assays in eWAT of 16-week-old male mice (n = 5). Multiple unpaired *t* test with the Holm–Sidak method. ∗*P* < 0.05; ∗∗*P* < 0.005. ; sWAT, subcutaneous WAT; eWAT, epididymal white adipose tissue; DEG, differentially expressed gene.

To further investigate the mechanisms behind CSN8 deletion-induced adipose tissue loss, we examined the expression of previously reported CSN targets. However, we found no significant changes in the expression of CHOP (14), p27 (31), or p21 (32) in the eWAT of 16-week-old Ctrl versus *Csn8*^*AKO*^ male mice (Supplemental Fig. 5A). Bulk RNA-Seq revealed reduced expression of oxidative stress-related glutathione metabolic genes (Fig. 6A). We also detected a 25% increase in the expression of antioxidant protein SOD2, but not catalase or glutathione peroxidase 4 (GPX4), in the eWAT of *Csn8*^*AKO*^ mice compared to Ctrl mice (Fig. 6B, E). These findings suggest that oxidative stress may be increased in adipose tissue of *CSN8*^*AKO*^ mice, as confirmed by increased protein carbonylation in eWAT (Fig. 6D, E). Destabilization of CSN causes excessive CUL neddylation, known to promote CRL-mediated ubiquitination (Fig. 2B). Surprisingly, the overall level of ubiquitination, as well as K48-linked polyubiquitination, the canonical signal for proteasomal degradation, decreased in the adipocytes isolated from *CSN8*^*AKO*^ eWAT (Fig. 6F, G). Such phenomena were recapitulated in the eWAT of Ctrl and *Csn8*^*AKO*^ mice (Supplemental Fig. S5B, C, respectively). Interestingly, the decreased ubiquitination signal was linked to increased proteasome activity, specifically trypsin-like activity in *Csn8*^*AKO*^ eWAT (Fig. 6H). These findings suggest that CSN8 deletion in WATs disrupts protein homeostasis by modulating protein ubiquitination and degradation.

### Mice with adipose-specific deletion of CSN8 exhibit brown fat atrophy and cold intolerance

**Fig. 7:**
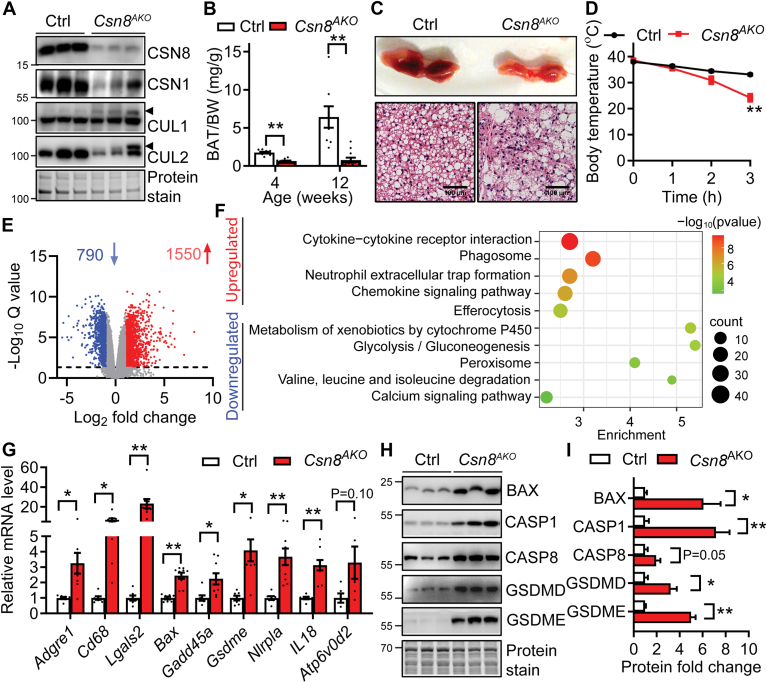
Adipose-specific deletion of CSN8 causes brown fat atrophy and cold intolerance by inducing apoptosis, pyroptosis, and inflammation. A: Western blot confirming the deletion of CSN8, the expression of CSN1, and neddylation of CULs in BAT. Arrowhead indicates neddylated CULs. B: BAT mass normalized to body weights (BW) in 4 and 12-week-old male Ctrl and *Csn8*^*AKO*^ mice. n = 8–11/group. Multiple unpaired *t* test with the Holm–Sidak method. C: Representative BAT morphology and histology. Scale bar = 50 μm. D: Body temperature in 12-week-old male Ctrl and *Csn8*^*AKO*^ mice after they were individually exposed to 4 °C for up to 3 h in the absence of food. n = 4–5/group. Multiple unpaired *t* test with the Holm–Sidak method. E: Volcano plot for differentially expressed genes (DEGs) in the BAT (n = 5). The *y*-axis denotes −log_10_*P*_*adj*._ (Q) values, while the *x*-axis shows log_2_ fold change (FC) values. DEGs; 1≤Log_2_FC ≤ −1. *P*_*adj*._<0.05. F: Bubble graph illustrates the upregulated and downregulated KEGG pathways filtered by DEGs in the BAT. The *x*-axis represents enrichment, while the color of the circle corresponds to the -log_10_*P* value, as indicated by the color bar. The size of the bubble represents the number of genes enriched in each pathway. Selective pathways indicated. G: RT-PCR of selective inflammatory, apoptotic and pyroptotic genes in BAT. n = 7–9/group. Multiple unpaired *t* test with the Holm-Sidak method. H–I: Western blot and quantification in BAT of Ctrl and *Csn8*^*AKO*^ mice. n = 5/group. Multiple unpaired *t* test with the Holm–Sidak method. All experiments were performed in the BAT of male 4-week-old Ctrl and *Csn8*^*AKO*^ mice if not specified. ∗*P* < 0.05; ∗∗*P* < 0.005. BAT, brown adipose tissue; KEGG, Kyoto Encyclopedia of Genes and Genomes.

We then examined whether deleting CSN8 also affects BAT mass and function. First, we confirmed the successful deletion of CSN8, which was accompanied by decreased CSN1 and increased neddylated CULs, such as CUL1 and CUL2, in *Csn8*^*AKO*^ BAT, suggesting disrupted CSN activity (Fig. 7A). Loss of BAT was more severe, with only 20% remaining in 4-week-old *Csn8*^*AKO*^ mice (Fig. 7B–C). By 12 weeks, BAT in *Csn8*^*AKO*^ mice failed to expand compared to control mice, indicating that loss of CSN8 causes BAT atrophy (Fig. 7B). The BAT of 4-week-old *Csn8*^*AKO*^ mice showed abnormal lipid accumulation and infiltration of inflammatory cells. The residual BAT of *Csn8*^*AKO*^ mice contained both small multilobular LDs and large unilocular LDs (Fig. 7C). Consistent with BAT atrophy and morphological abnormalities, *Csn8*^*AKO*^ mice were extremely cold-sensitive during acute cold exposure compared to controls (Fig. 7D). Bulk RNA-Seq of BAT identified 790 genes that were significantly downregulated and 1550 genes that were notably upregulated in *Csn8*^*AKO*^ BAT (Fig. 7E). KEGG analysis revealed similar enriched pathways as seen in the sWAT of *Csn8*^*AKO*^ mice, including cytokine-cytokine receptor interaction, chemokine signaling, neutrophil extracellular trap formation, as well as phagosome and efferocytosis pathways (Fig. 7F). Conversely, genes involved in the cytochrome P450 pathway, glycolysis/gluconeogenesis, peroxisomal functions, amino acid metabolism, and calcium signaling were significantly downregulated in BAT of *Csn8*^*AKO*^ mice (Fig. 7F). RT-PCR confirmed increased expression of inflammatory markers such as *Adgre1*, *Cd68*, and *Lgals3* in BAT of *Csn8*^*AKO*^ mice (Fig. 7G). Heatmaps further showed significant upregulation of genes related to apoptosis, cytosolic DNA signaling, phagosome, and efferocytosis, indicating the presence of apoptosis and pyroptosis in CSN8-deficient BAT (Supplemental Fig. S6). The upregulation of selected apoptotic and pyroptotic genes was further confirmed by RT-PCR (Fig. 7G) and western blot (Fig. 7H–I), clearly demonstrating that CSN8-deficient BAT exhibits apoptosis and pyroptosis. Overall, these findings underscore the crucial role of the CSN8-mediated COP9 complex in maintaining BAT mass and thermogenesis.

### *Csn8*^*AKO*^ mice are protected from HFD-induced adipose mass gain but are more susceptible to hepatic steatosis and insulin resistance

**Fig. 8:**
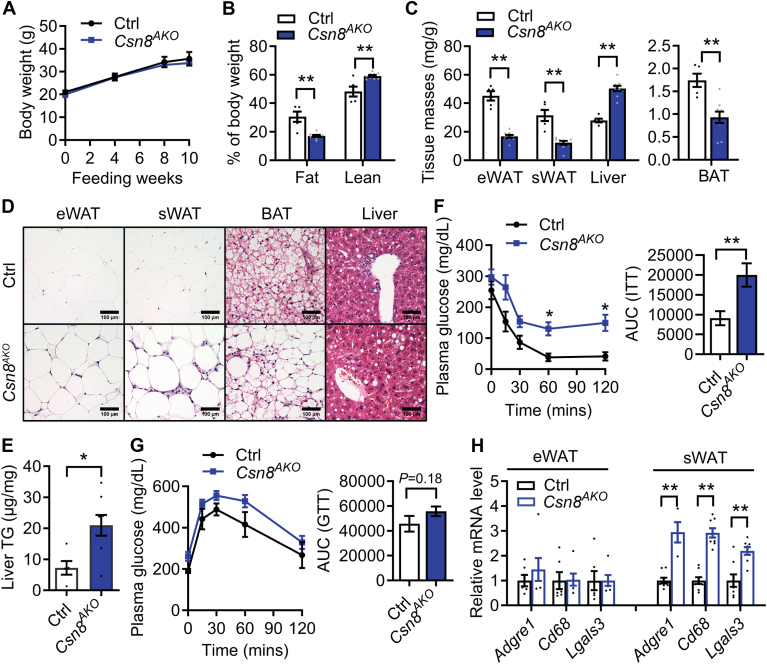
*Csn8*^*AKO*^ mice are protected from high-fat diet-induced obesity but are susceptible to adipose inflammation, hepatic steatosis, and insulin resistance. 6-week-old male Ctrl and *Csn8*^*AKO*^ mice were fed with a 60% high-fat diet (HFD) for up to 10 weeks (A) growth curve; (B) % of fat and lean masses; (C) eWAT, sWAT, and liver weights as normalized to body weights. Multiple unpaired *t* test with the Holm–Sidak method. D: Representative hematoxylin & eosin staining of eWAT, sWAT, and liver. Scale bar = 50 μm. E: Liver triglyceride contents normalized to tissue mass. Unpaired *t* test. F: Insulin tolerance tests and area under the curve (AUC). G: Glucose tolerance tests and AUC. H: RT-PCR of selective inflammatory genes in eWAT and sWAT of 16-week-old Ctrl and *Csn8*^*AKO*^ male mice fed with HFD. n = 6–9/group. Multiple unpaired *t* test with the Holm–Sidak method. ∗*P* < 0.05; ∗∗*P* < 0.005. ; sWAT, subcutaneous WAT; eWAT, epididymal white adipose tissue.

To investigate the role of adipocyte CSN8 in overnutrition, *Csn8*^*AKO*^ mice were fed an HFD for 10 weeks. We found that *Csn8*^*AKO*^ and Ctrl mice had similar BWs (Fig. 8A). However, body composition analysis showed a 43% decrease in total body fat mass (Fig. 8B), with eWAT, sWAT, and BAT decreased by 63%, 60% and 46%, respectively, in *Csn8*^*AKO*^ mice (Fig. 8C). In contrast, HFD-fed *Csn8*^*AKO*^ mice showed a 23% increase in lean mass (Fig. 8B) and a 1.8-fold increase in liver weight (Fig. 8C). There were no significant differences in fasting blood glucose, TGs, cholesterol, or NEFA levels between groups, although fasting glycerol levels were again lower in HFD-fed *Csn8*^*AKO*^ mice (Table 1). Histological analysis showed that HFD increased the number of infiltrating cells in eWAT and, more notably, in sWAT of *Csn8*^*AKO*^ mice. BAT of *Csn8*^*AKO*^ mice showed whitening phenotype as compared to Ctrl mice under HFD (Fig. 8D). Furthermore, more LDs were observed in the livers of HFD-fed *Csn8*^*AKO*^ mice (Fig. 8D), which was confirmed by exacerbated TG deposition, indicating worsened hepatic steatosis caused by HFD (Fig. 8E). Unsurprisingly, HFD-fed *Csn8*^*AKO*^ mice were not protected from metabolic disorders, displaying worsened insulin resistance and a trend toward glucose intolerance compared to HFD-fed Ctrl mice (Fig. 8F, G). RT-PCR showed a more pronounced increase in the expression of inflammatory marker genes in sWAT, but not eWAT, of HFD-fed *Csn8*^*AKO*^ mice. Overall, these findings suggest that CSN8 deletion decreases adipose tissue accumulation but promotes hepatic steatosis and insulin resistance during diet-induced obesity.

## Discussion

In this study, we demonstrate that CSN8 and the CSN complex are essential for maintaining adipose tissue mass and overall metabolic health. Loss of CSN8 disrupted the CSN complex formation and abrogated the deneddylation of CULs. However, it did not perturb white adipogenesis in vitro. Instead, deletion of CSN8 in mature adipocytes causes significant loss of both white and brown adipose tissues in mice, leading to adipose inflammation, insulin resistance, and altered energy consumption and expenditure. Furthermore, adipocyte death is increased in conjunction with COP9 signalosome deficiency, in association with altered protein homeostasis. These findings collectively support the essential functional role of CSN8/CSN in maintaining adipose homeostasis in vivo.

CSN and its variants CSN^CSN7A^ and CSN^CSN7B^ have been shown to influence adipogenesis (11, 12). During adipocyte differentiation, CSN subunits show different regulation patterns, with CSN6/8 levels decreasing and CSN7 levels increasing in mature adipocytes compared to preadipocytes (13). Knocking down CSN1 or CSN7A was reported to impair adipogenesis (12, 14, 15); conversely, overexpressing CSN7A promoted adipogenesis (16). In contrast, our study found no significant change in CSN8 expression between mouse and human adipose progenitors and mature adipocytes. Moreover, even with an 80%–90% knockdown of CSN8, adipogenesis was unaffected. These discrepancies may stem from differences in cell types or the use of different knockdown techniques. Further research, including in vivo experiments with CSN8 deletion in adipose progenitors and in vitro experiments using different cell types, is necessary to clarify the role of CSN8/CSN in adipogenesis and the regulation of fat mass.

By conditionally deleting the *Csn8* gene in mouse adipocytes, we demonstrate for the first time that CSN is essential for maintaining both white and brown adipocyte survival and adipose tissue mass. These physiological functions are consistent across sexes, as both male and female *Csn8*^*AKO*^ mice experienced similar fat loss and metabolic dysfunction. The increased lean mass in *Csn8*^*AKO*^ mice could be attributed to hormonal and metabolic adaptations, such as leptin-induced hyperphagia and insulin resistance, which drive nutrient allocation to organ growth, as reported in lipodystrophy mice (25). Notably, sWAT and BAT declined more rapidly and to a greater extent than eWAT in chow-fed *Csn8*^*AKO*^ mice. Under a HFD, *Csn8*^*AKO*^ mice exhibited a similar loss of eWAT, sWAT, and BAT, with more pronounced inflammation observed in sWAT. The reasons for depot- and diet-specific effects of CSN8 deletion are unclear but may involve crosstalk between WAT and BAT, as well as distinct CSN-regulated pathways essential for maintaining eWAT, sWAT, and BAT. Interestingly, in the presence of BAT atrophy, the overall energy expenditure in *Csn8*^*AKO*^ mice tends to be higher. We deduce that such changes may be due to leptin deficiency, which causes hyperphagia, a condition known to trigger diet-induced thermogenesis (33). Interestingly, despite insulin resistance, *Csn8*^*AKO*^ mice failed to develop overt glucose intolerance, even when challenged with a HFD. Such phenomena could be attributed to compensatory mechanisms, such as beta-cell hyperplasia and hyperinsulinemia, which warrant further investigation in our *Csn8*^*AKO*^ mice. Meanwhile, the mechanisms underlying a whitening phenotype in BAT of HFD-fed *Csn8*^*AKO*^ mice are interesting. It remains to be investigated whether the effects are due to lipid shunting from limited storage in the WAT of *Csn8*^*AKO*^ mice or an inherent brown adipocyte-specific loss of CSN8. Future studies should examine how diet interacts with CSN8 deficiency to influence the BAT phenotype and energy expenditure in these mice. Overall, our findings highlight the crucial role of CSN8/CSN in supporting adipose tissue mass under both normal conditions and in diet-induced obesity.

Our data demonstrates, for the first time, the essential role of the CSN8/CSN complex in regulating adipose tissue apoptosis and pyroptosis. In *Saccharomyces cerevisiae*, defects in CSN assembly or activity cause disrupted lipid homeostasis and endoplasmic reticulum (ER) stress (34). However, CSN-deficient adipose tissue does not demonstrate ER stress, suggesting that ER stress is not the mechanism that causes adipocyte death. In obese mouse models, pyroptosis-related proteins, such as NLRP3, CASP1, and IL-1 cytokines, are elevated in both adipocytes and macrophages (35). In *Csn8*^*AKO*^ mice, the increase in pyroptosis markers is primarily observed in nonadipocyte stromal vascular fractions of CSN8-deleted eWAT but not sWAT. Notably, it was challenging to detect the cleaved isoforms of apoptosis and pyroptosis marker proteins in *Csn8*^*AKO*^ BAT, despite an even more overt upregulation of apoptotic and pyroptotic gene expressions. In addition, even though the hallmark inflammasome marker genes were upregulated, there were no consistent increases in IL-18 or IL-1β in CSN8-deleted white and brown adipocytes. These discrepancies may reflect depot-specific differences in cell death. The presence of distinct forms of cell death in specific cell types in CSN8-deleted adipose tissue calls for further elucidation using fluorescence-activated cell sorting and/or electron microscopy. Notably, adipocytes differentiated in vitro without CSN8 did not undergo apoptosis or pyroptosis. It remains unclear whether compensatory mechanisms are activated during early differentiation when CSN8 is absent. However, our findings indicate that *Csn8*^*AKO*^ adipocytes may create a damaging inflammatory environment that leads to the death of adipocytes and SVCs in vivo. Dying cells then further release damage-associated molecular patterns, which can activate phagocyte microbicidal oxidase systems, leading to the production of reactive oxygen species and exacerbating inflammation and cell death (36). In addition, we observed increased efferocytosis and phagosome activity associated with oxidative stress and inflammation in CSN8-deleted fat tissue. These observations underscore the essential role of CSN8/CSN in maintaining adipocyte survival and facilitating adipose tissue remodeling.

Notably, deletion of CSN8/CSN causes end-stage heart failure and is associated with impaired proteasome activities (18, 37). However, in our study, when examining the eWAT of *Csn8*^*AKO*^ mice at the early stage of fat loss, the total ubiquitination and K48-linked ubiquitination in CSN8-deleted adipocytes and tissues were not increased; rather, they were decreased. This is inconsistent with the canonical role of CSN8/CSN in regulating CRL activities and protein ubiquitination. Interestingly, such changes are associated with increased proteasome activity in CSN8-deleted adipose tissues. Since K48-linked ubiquitination typically targets proteins for proteasomal degradation, we speculate that increased proteasome activity may contribute to reduced ubiquitination. However, the exact molecular mechanisms linking decreased ubiquitination signals to the elevated trypsin-like proteasome activity in this context require further research. Taken together, the discrepancies observed in how CSN deletion impacts UPS may be organ-specific or influenced by disease progression.

Our animal model of adipocyte-specific deletion of CSN8 enhances understanding of the pathophysiological significance of adipose UPS disruption in the development of adipose impairment and insulin resistance. Proteasome activity is decreased in adipose tissue under insulin-resistant conditions (38), and increased proteasome activities mediated by NRF1 are essential in adaptive thermogenesis (39). It remains unknown whether the heightened proteasome activities in the insulin-resistant CSN8-deleted WATs are a direct or indirect consequence of CSN8 deletion, since CSN is functionally related to the ubiquitin (Ub)/26S proteasome system based on its structural similarity to, and copurification with, the 26S proteasome lid (40). Bulk RNA-sequencing identified no differences in the mRNA expression of proteasome subunits (data not shown). Because CSN8/CSN influences protein expression via posttranslational mechanisms, RNA-Seq alone cannot directly pinpoint CSN8/CSN targets. Future studies should use quantitative proteomics to identify potential targets and precisely elucidate the underlying mechanisms.

In summary, our study shows, for the first time, that CSN8/CSN is essential for the survival of mature adipocytes and for the remodeling of fat tissue. The absence of CSN8/CSN leads to adipocyte apoptosis and pyroptosis via perturbing protein homeostasis, inducing oxidative stress, inflammation, and insulin resistance, ultimately disrupting metabolic homeostasis.

## Data availability

All RNA-Seq data are submitted to the GEO database (GSE314831). All other data will be made available upon request to the corresponding author.

## Supplemental data

This article contains supplemental data.

## Conflict of interest

The authors declare that they have no conflicts of interest with the contents of this article.
