# Supplementary material for: COP9 signalosome is required for adipose tissue maintenance and metabolic health
Source: J Lipid Res. 2026 Jan 8;67(2):100977. doi: 10.1016/j.jlr.2026.100977 (PMC12887410; doi:10.1016/j.jlr.2026.100977)
Supplement: Supplementary Material [file mmc1.docx]

**Supplemental Data**

**COP9 signalosome is Required for Adipose Tissue Maintenance and Metabolic Health**

Hongyi Zhou, Shayantani Chakraborty, Xuelei Zhao, Neal L Weintraub, Huabo Su, Weiqin Chen

**Supplemental Table 1. List of primers for murine genes used in the present study**

| **Gene name** | **5’ forward primer sequence** | **3’ reverse primer sequence** |
| --- | --- | --- |
| *Adgre1* | CTTTGGCTATGGGCTTCCAGTC | GCAAGGAGGACAGAGTTTATCGTG |
| *Atp6v0d2* | TTCTGCAAGGATCACGGTGA | AGCCAGGAAGTTGCCATAGT |
| *Bax* | TGTTTGCTGATGGCAACTTC | GGAGGAAGTCCAGTGTCCAG |
| *Cd68* | CCAATTCAGGGTGGAAGAAA | GACTGTACTCGGGCTCTGATG |
| *Gadd45a* | CTGCTGCTACTGGAGAACGA | GCAGGATCCTTCCATTGTG |
| *Gsdme* | GGCAGAGACCCAAGTACCAG | CCTTTGCCACCAACGTTCAG |
| *Il18* | ACTTTGGCCGACTTCACTGT | TTCACAGAGAGGGTCACAGC |
| *Il-1β* | CTGCTTCCAAACCTTTGACC | CTTCTCCACAGCCACAATGA |
| *Lgals3* | CCTATGACCTGCCCTTGC | CCCAGTTGGCTGATTTCC |
| *Lipa* | ATGGGAAACAGCAGAGGAAA | CTATGGTGCAGCCTTGAGAA |
| *Nlrp1a* | AATGGGAGGCCTTGCTGAAA | ATGCTGGTCCATGAAGTGCA |
| *Plin1* | CACCTGCGGCTGTGCTGG | CGATGTCTCGGAATTCGCT |
| *Ppara* | CCACGAAGCCTACCTGAAGA | ACTGGCAGCAGTGGAAGAAT |
| *Pparγ2* | TCTCCTGTTGACCCAGAGCA | GTGGAGCAGAAATGCTGGAG |
| *Ppia* | CTGTTTGCAGACAAAGTTCCA | AGGATGAAGTTCTCATCCTCA |
| *Rplp0* | CGCTTTCTGGAGGGTGTCCGC | TGCCAGGACGCGCTTGTACC |
| *Tnfα* | TGCCTATGTCTCAGCCTCTTC | GGAGGCCATTTGGGAACT |

**Supplemental Table 2.** Antibodies or reagents used for immunoblotting, immunohistochemistry, and immunofluorescent staining.

| **Antibody** | **Supplier (Catalog No.)** | **RRID** |
| --- | --- | --- |
| AKT | Cell signaling technology (9272) | RRID:AB_329827 |
| Phospho-AKT (ser473) | Cell signaling technology (4060) | RRID:AB_2315049 |
| BAX | Proteintech (50599-2-Ig) | RRID:AB_2061561 |
| CASP1 (E9R2D) | Cell signaling technology (83383) | RRID:AB_3662792 |
| Cleaved CASP1 (Asp296) (E2G2I) | Cell signaling technology (89332) | RRID:AB_2923067 |
| CASP3 | Cell signaling technology (9662) | RRID:AB_331439 |
| Cleaved CASP3 (Asp175) | Cell signaling technology (9661) | RRID:AB_2341188 |
| CASP8 (D35G2) | Cell signaling technology (4790) | RRID:AB_10545768 |
| Cleaved CASP8 (Asp387) | Cell signaling technology (9429) | RRID:AB_2068300 |
| Catalase | Proteintech (21260-1-AP) | RRID:AB_10733099 |
| CHOP (L63F7) | Cell signaling technology (2895) | RRID:AB_2089254 |
| CSN1 | Enzo Life Sciences (BML-PW8285-0025) | RRID:AB_2051159 |
| CSN8 | Enzo Life Sciences (BML-PW8290-0025) | RRID:AB_2051167 |
| CUL1 | Proteintech (12895-1-AP) | RRID:AB_2086291 |
| CUL2 | Abcam (ab166917) | RRID:AB_3411917 |
| CUL4A | Proteintech (14851-1-AP) | RRID:AB_2261175 |
| GAPDH | Proteintech (60004-1-IG) | RRID:AB_2107436 |
| GPX4 (EPNCIR144) | Abcam (ab125066) | RRID:AB_10973901 |
| GSDMD (E9S1X) | Cell signaling technology (39754) | RRID:AB_2916333 |
| Cleaved GSDMD (Asp276) (E3E3P) | Cell signaling technology (10137) | RRID:AB_2923068 |
| GSDME (E1C5B) | Cell signaling technology (88874) |  |
| MAC2 | Biolegends (125401) | RRID: AB_1134237 |
| NLRP3 (D4D8T) | Cell signaling technology (15101) | RRID:AB_2722591 |
| P21/CDKN1A (EPR3993) | Abcam (ab109199) | RRID:AB_10861551 |
| P27Kip1 (SX53G8.5)/CDKN1B | Cell signaling technology (3698) | RRID:AB_2077832 |
| PLIN1 | Cell signaling technology (9349) | RRID:AB_10829911 |
| PPARγ | Cell signaling technology (2435) | RRID:AB_2166051 |
| SOD2 | Proteintech (24127-1-AP) | RRID:AB_2879437 |
| Ubiquitin (P4D1) | Cell signaling technology (3936) | RRID:AB_331292 |
| K48-linked-Ubiquitin | Cell signaling technology (8081) | RRID:AB_10859893 |

**
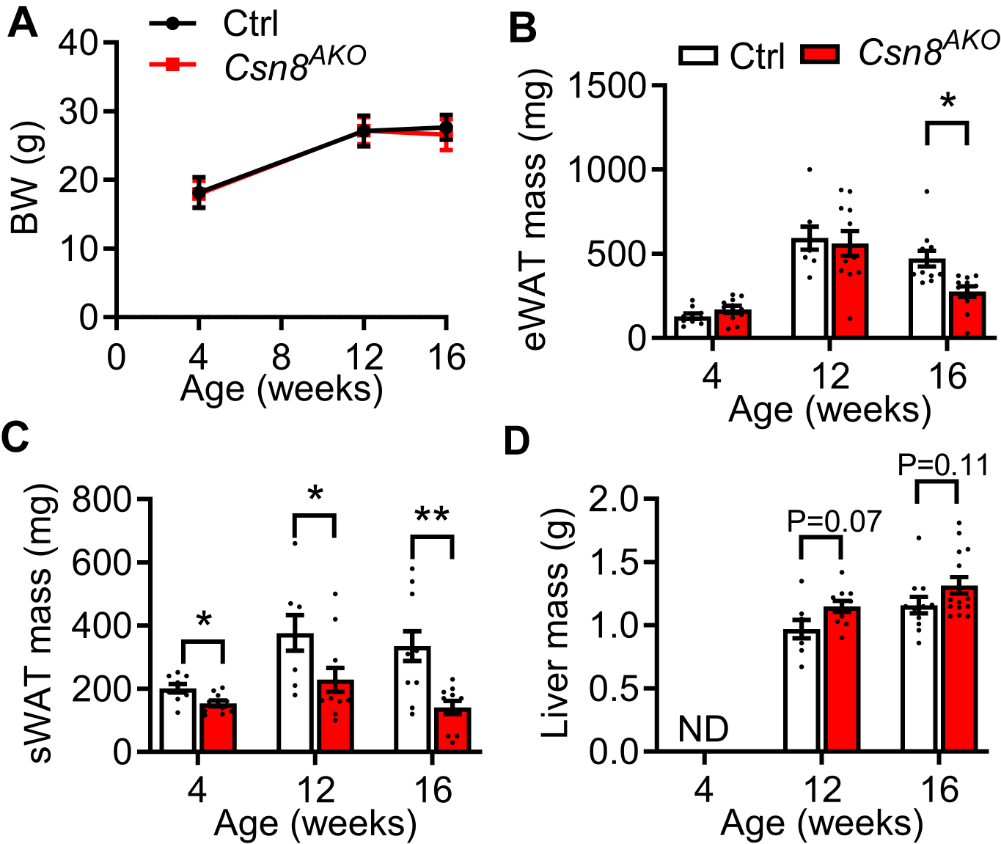
**

**Supplemental Figure S1. Adipocyte-specific loss of CSN8 reduces adiposity in male mice.**

(**A**) Growth curve up to 16 weeks of age in male Ctrl and *Csn8^AKO^* mice. n=8-11/group. (**B-D**) Absolute masses of (**B**) epididymal white adipose tissue (eWAT), (**C**) subcutaneous WAT (sWAT), and (**D**) liver weights in 4-, 12-, and 16-week-old male Ctrl and *Csn8^AKO^* mice. n=8-15/group. ND: not determined. n = 6-13/group. Multiple unpaired t-test with the Holm-Sidak method. *P<0.05; **P<0.005 versus Ctrl.


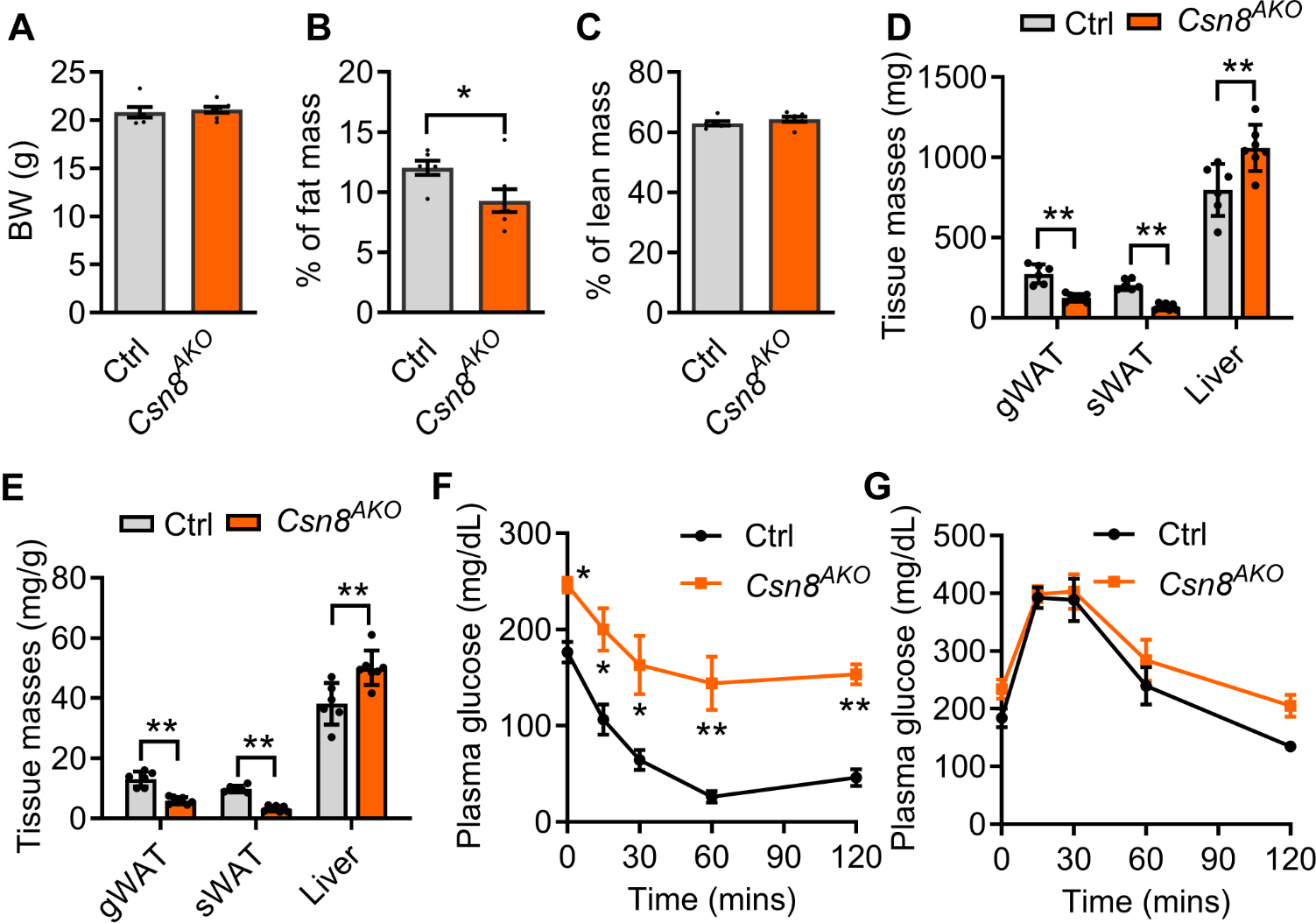


**Supplemental Figure S2. Adipocyte-specific loss of CSN8 reduces adiposity and perturbs metabolic homeostasis in female mice.** (**A**) Body weights (BW), (**B**) % of fat mass, (**C**) % of lean mass. Unpaired t-test. (**D**) Absolute masses of gonadal white adipose tissue (gWAT), subcutaneous WAT (sWAT), and liver weights and (**E**) tissues masses after normalization to body weights. (**F**) Insulin tolerance tests and (**G**) glucose tolerance tests. Multiple t-tests with the Holm-Sidak method. 16-week-old female Ctrl and *Csn8^AKO^* mice were used. n = 6-7/group. *P<0.05; **P<0.005.

**
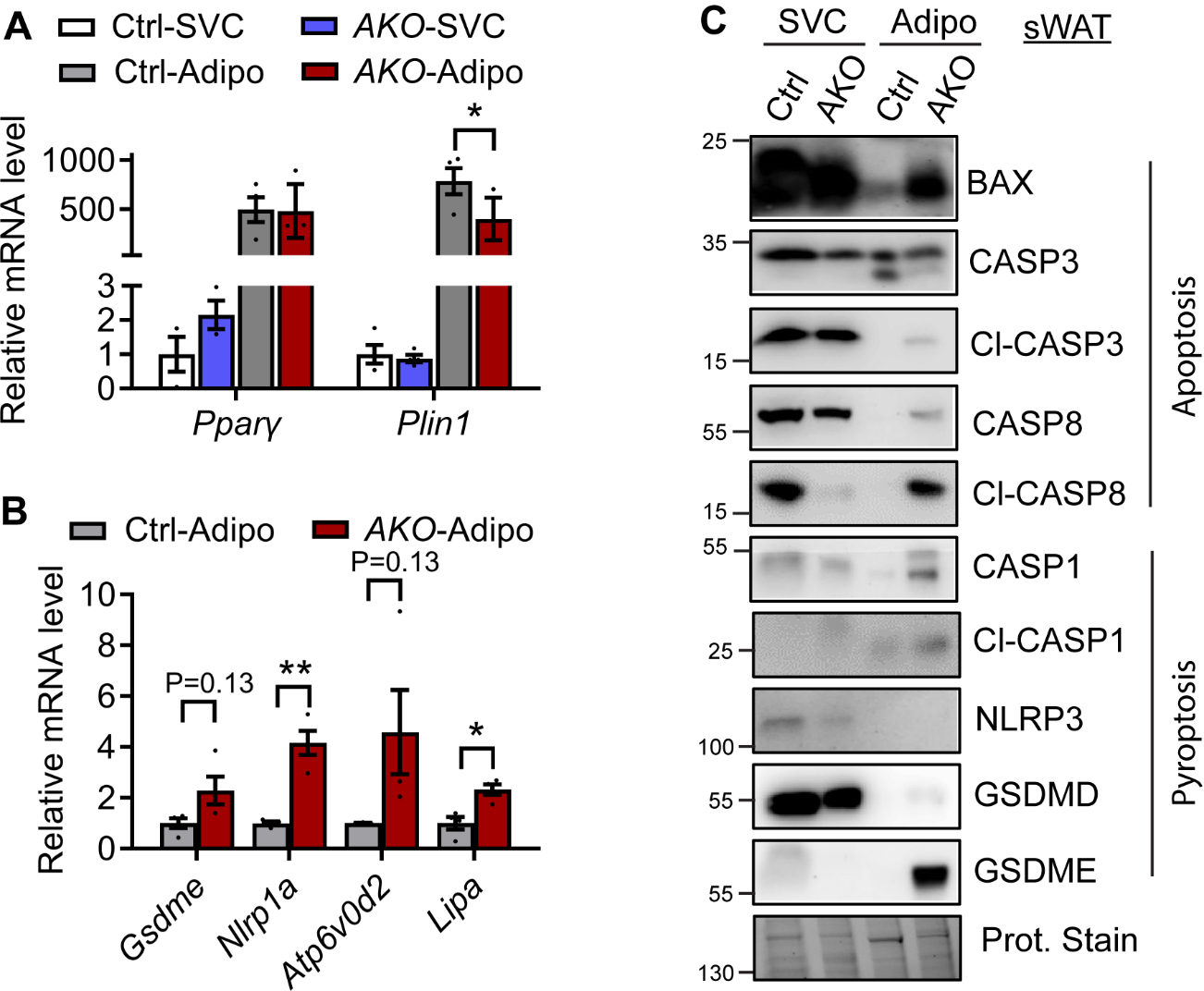
**

**Supplemental Figure S3. Adipocyte-specific loss of CSN8 induced apoptosis and pyroptosis in subcutaneous adipose tissue.**

sWAT from 16-week-old control (Ctrl) and *Csn8^AKO^* (AKO) male mice was fractionated into stromal vascular cells (SVC) and adipocytes (Adipo) fractions. (**A**) The expression of adipocyte marker genes (*Pparγ*, *Plin1*) was confirmed by RT-PCR. n = 4/group, with each group combined from 3 mice. (**B**) The expression of selective genes involved in cytosolic DNA sensing (*Gsdme*, *Nlrp1a*), phagosome (*Atp6v0d2*), and efferocytosis (*Lipa*) was confirmed in adipocyte fractions, with the gene expression in Ctrl-Adipo normalized to 1. Multiple t-tests with the Holm-Sidak method. (**C**) Western blot in SVC and Adipo fractions from sWAT of 16-week-old male Ctrl and *Csn8^AKO^* mice. Each sample is combined from three animals. Representatives of two independent experiments were shown. *P<0.05; **P<0.005.


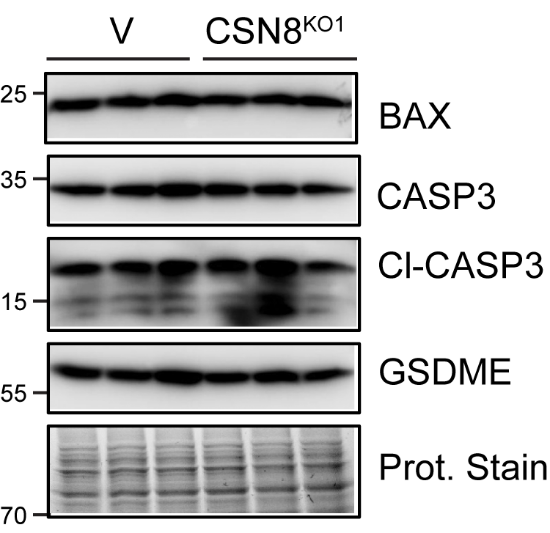


**Supplemental Figure S4. Protein expression in differentiated Ctrl and CSN8-deleted 3T3-L1 adipocytes.** 3T3-L1 preadipocytes were infected with lentiviruses expressing empty vector (V) or gRNA1 against the murine *Csn8* gene to knock down CSN8 (KO1). Cells were differentiated using a standard protocol, and protein expression was examined in D8 mature adipocytes**.**


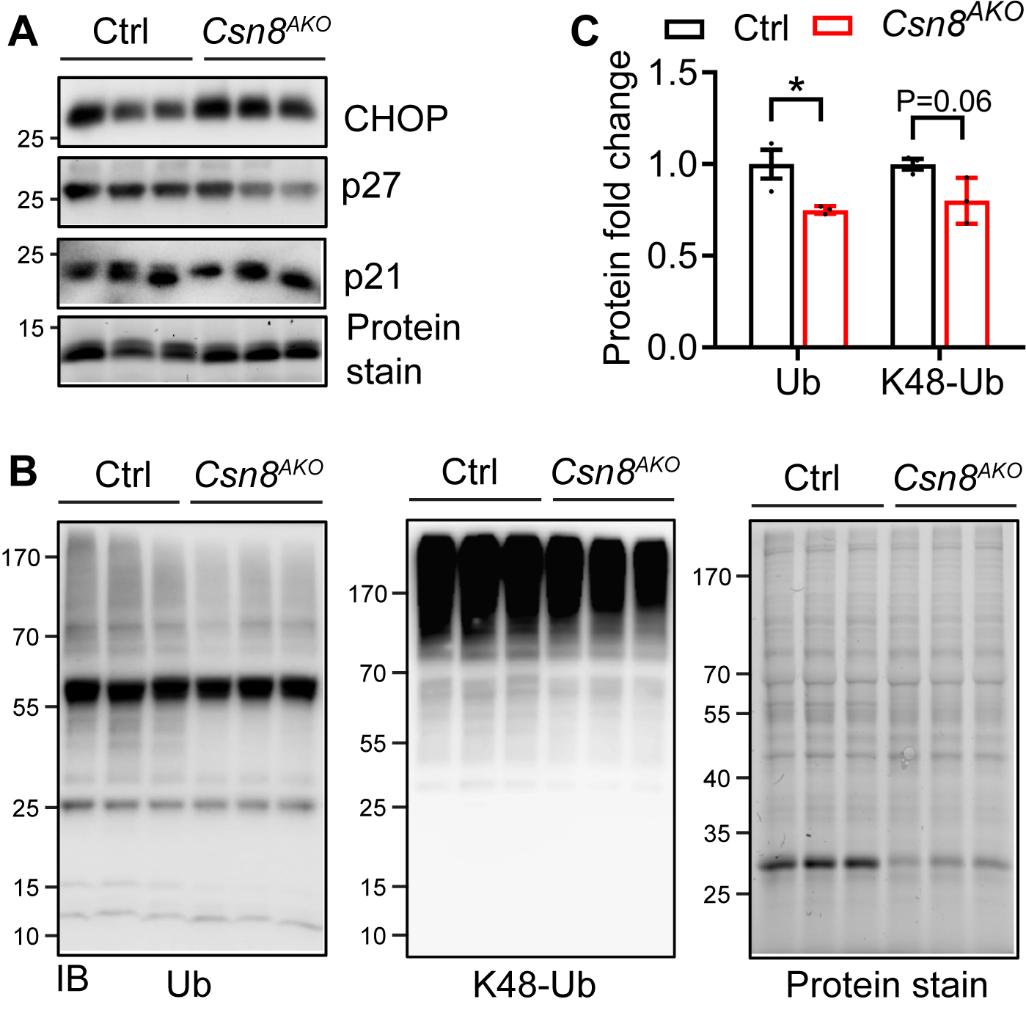


**Supplemental Figure S5.** **CSN8 deletion reduces protein ubiquitination in adipose tissue.**

(**A-B**) Western blot of CSN targeted proteins, ubiquitinated and K48-ubiquitinated proteins, respectively. (**C**) Quantitative analyses of ubiquitinated and K48-ubiquitinated proteins after normalization to the protein-stained signals. eWAT of 16-week-old male Ctrl and *Csn8^AKO^* mice were used (n = 3). Unpaired t-test. *P<0.05; **P<0.005.


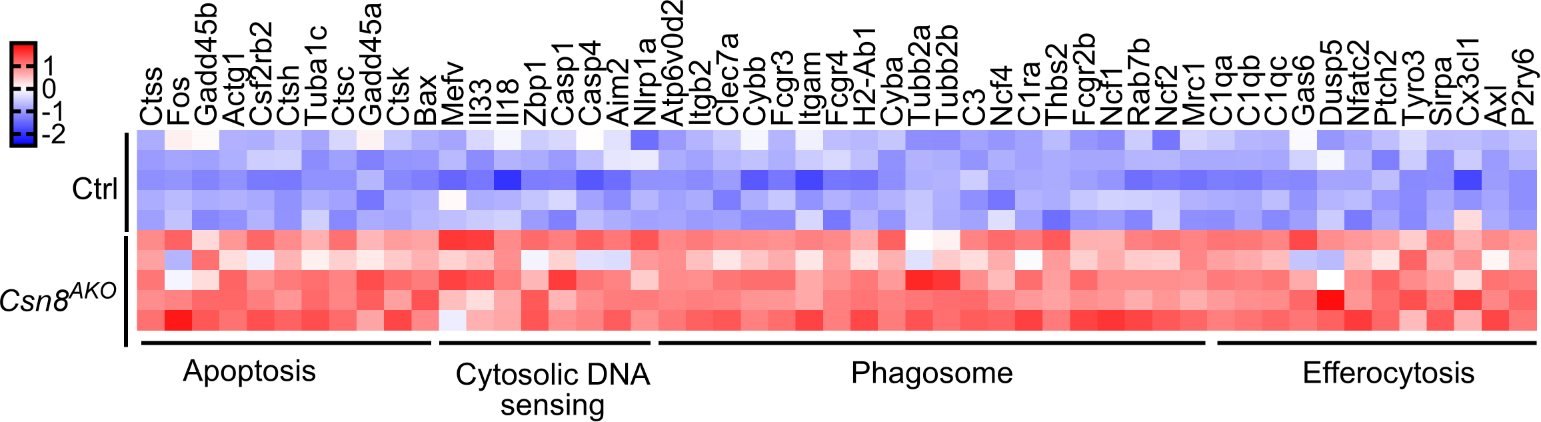


**Supplemental Figure S6.** Heatmaps showing the differential expression of genes involved in apoptosis, cytosolic DNA sensing, phagosome and efferocytosis in the BAT of 4-week-old Ctrl and *Csn8^AKO^* male mice (n = 5) (DEGs cut-off: 1≤Log_2_FC≤-1, *P_adj._*<0.05).
